# Biofilm Development on *Caenorhabditis elegans* by *Yersinia* Is Facilitated by Quorum Sensing-Dependent Repression of Type III Secretion

**DOI:** 10.1371/journal.ppat.1001250

**Published:** 2011-01-06

**Authors:** Steve Atkinson, Robert J. Goldstone, George W. P. Joshua, Chien-Yi Chang, Hannah L. Patrick, Miguel Cámara, Brendan W. Wren, Paul Williams

**Affiliations:** 1 School of Molecular Medical Sciences, Centre for Biomolecular Science, University of Nottingham, Nottingham, United Kingdom; 2 Department of Infectious and Tropical Diseases, London School of Hygiene and Tropical Medicine, London, United Kingdom; Massachusetts General Hospital and Harvard Medical School, United States of America

## Abstract

*Yersinia pseudotuberculosis* forms biofilms on *Caenorhabditis elegans* which block nematode feeding. This genetically amenable host-pathogen model has important implications for biofilm development on living, motile surfaces. Here we show that *Y. pseudotuberculosis* biofilm development on *C. elegans* is governed by *N-*acylhomoserine lactone (AHL)-mediated quorum sensing (QS) since (i) AHLs are produced in nematode associated biofilms and (ii) *Y. pseudotuberculosis* strains expressing an AHL-degrading enzyme or in which the AHL synthase (*ypsI* and *ytbI)* or response regulator *(ypsR* and *ytbR)* genes have been mutated, are attenuated. Although biofilm formation is also attenuated in *Y. pseudotuberculosis* strains carrying mutations in the QS-controlled motility regulator genes, *flhDC* and *fliA*, and the flagellin export gene, *flhA*, flagella are not required since *fliC* mutants form normal biofilms. However, in contrast to the parent and *fliC* mutant, Yop virulon proteins are up-regulated in *flhDC*, *fliA* and *flhA* mutants in a temperature and calcium independent manner. Similar observations were found for the *Y. pseudotuberculosis* QS mutants, indicating that the Yop virulon is repressed by QS via the master motility regulator, flhDC. By curing the pYV virulence plasmid from the ypsI/ytbI mutant, by growing YpIII under conditions permissive for type III needle formation but not Yop secretion and by mutating the type III secretion apparatus gene, yscJ, we show that biofilm formation can be restored in flhDC and ypsI/ytbI mutants. These data demonstrate that type III secretion blocks biofilm formation and is reciprocally regulated with motility via QS.

## Author Summary

Many Gram-negative bacteria communicate by producing and sensing the presence of chemical signal molecules such as the *N-*acylhomoserine lactones (AHLs). Bacterial cells use AHLs to convey information about their environment, metabolism and population size. This type of chemical signalling is called ‘quorum sensing’ (QS) and is often used by pathogenic bacteria to promote acute or chronic infections through the control of motility, toxins, tissue degrading enzymes and surface-associated biofilms. *Yersinia pseudotuberculosis* is a human pathogen which forms biofilms on the surface of the nematode worm, *Caenorhabditis elegans.* This offers a simple means for investigating biofilm development on living tissues and can be used to identify genetic features of both the pathogen and the host that contribute to biofilm-associated infections. We have discovered that quorum sensing is required for *Y. pseudotuberculosis* biofilm formation on *C. elegans* through a regulatory pathway which involves the master motility regulator protein (FlhDC) reciprocally controlling bacterial swimming and the construction of a specialized secretion needle that delivers proteins into mammalian cells to disrupt their normal activities.

## Introduction

The human pathogenic *Yersiniae (Yersinia pseudotuberculosis*, *Yersinia enterocolitica* and *Yersinia pestis)* share a high degree of DNA identity, but cause distinct diseases ranging from enterocolitis (*Y. enterocolitica* and *Y. pseudotuberculosis*) to pneumonic, bubonic or septicaemic plague (*Y. pestis*). Essential for the virulence of all pathogenic *Yersiniae*, is the ∼70-kb pYV virulence plasmid, which encodes the Yop virulon. This consists of a type III secretion system which enables *Yersinia* to inject multiple Yop effector proteins directly into the cytosol of eukaryotic cells and so subvert host cell signalling pathways (for reviews see [1]–[3]. Yop virulon genes are tightly regulated by environmental conditions and in particular, temperature (only expressing at 37°C) and Ca^2+^ concentration (reviewed in [4]).

*Y. pestis* and *Y. pseudotuberculosis* are capable of forming biofilms around the anterior and along the surface of the nematode *Caenorhabditis elegans* [5], [6]. However, biofilm formation is strain-dependent and a study of over 40 different *Y. pseudotuberculosis* strains showed that some formed biofilms on *C. elegans* but not on abiotic polystyrene surfaces and vice versa [6]. No relationship was observed between strains forming biofilms on *C. elegans* and those that formed biofilms on polystyrene surfaces. These findings suggest that biofilm development on the living surface of *C. elegans* is different from that on an abiotic surface such as polystyrene.

*Y. pestis* is transferred between mammalian hosts by a flea borne vector that feeds on blood. The *hmsHFRS* operon is key to the colonisation and blockage of the flea proventriculus which results from the accumulation of biofilm [7]–[9] and *hmsHFRS* mutants of both *Y. pestis* and *Y. pseudotuberculosis* fail to form biofilms on *C. elegans*. Since *C. elegans* has been thoroughly studied at the genetic level and orthologous genes frequently studied in human health and disease, the *C. elegans/Yersinia* model can be used to identify genetic features of both the pathogen and the host that contribute to biofilm-mediated interactions between bacteria and invertebrates. These in turn have interesting implications for both the *Yersinia*/flea and human biofilm-centred infections. Although there are some limitations, the importance of *C. elegans* as a model organism for investigating prokaryotic/eukaryote interactions should not be overlooked given that nematodes are the most abundant animals on the Earth [10].

Although *Y. pseudotuberculosis* does not readily colonise fleas, biofilm formation may alternatively be involved in the prevention of predatory feeding as has been noted for other soil bacteria [11]. Whether the bacteria-invertebrate biofilm relationship is bacterially driven or is a two way interactive process between the bacteria and nematode is not fully understood. It has however been postulated that nematodes accumulate the bacterially derived extracellular matrix (ECM) passively by virtue of their movement through a lawn of bacteria [12] and there is evidence to show that biofilms do not accumulate on the surface of non-motile *C. elegans*. This implies that a prerequisite for biofilm formation is nematode translocation which provides the necessary contact between bacteria and nematode [12]. However, *Y. pseudotuberculosis* is unable to form biofilms on a number of motile *C. elegans* mutants such as *srf-2*, *srf-3* and *srf-5* [6] and *bah-1*, *bah-2* and *bah-3*. Conversely many natural strains of *Y. pseudotuberculosis* fail to form biofilms on *C. elegans* as do a number of *Y. pseudotuberculosis* strains with mutations in lipopolysaccharide biosynthesis, signal transduction and *hms* genes [6]. Such findings imply the existence of an adaptive interaction between the nematode and the bacterium rather than simply the passive adherence of bacterially derived ECM [6].

Bacteria possess multiple integrated sensory systems that govern adaptation to environmental challenges including the local cell population density. Such population-dependent adaptive behaviour often takes the form of perception and processing of chemical information and is termed quorum sensing (QS). For many Gram negative bacteria this involves the use of self-generated diffusible signal molecules such as the *N*-acyl homoserine lactones (AHLs). These are usually synthesised and sensed *via* members of the LuxI AHL synthase and LuxR response regulator protein families respectively. QS enables bacteria to determine, by monitoring the concentration of a signal molecule, when the number of individuals in the population are sufficient (a quorum) to make a collective ‘decision’ to alter their behaviour in response to environmental challenges [13]–[16]. Such behavioural decisions impact on bacterial motility, secondary metabolism, virulence, and biofilm development [17].

*Y. pseudotuberculosis* produces four major AHLs via a QS system consisting of two genetic loci termed *ypsR*/*ypsI* and *ytbR*/*ytbI* which control cell aggregation/flocculation and swimming motility [18], [19]. This system is organized hierarchically with YpsR and its cognate AHLs regulating *ytbR* and *ytbI* as well as *ypsR* and *ypsI*. The YpsR/YpsI and YtbR/YtbI QS system in turn fine tunes swimming motility by governing the expression of two key regulators of the motility cascade, namely *flhDC* and *fliA* [19]. AHL-dependent QS also controls motility in *Y. enterocolitica* [20] *Y. pestis* produces a similar range of AHLs to *Y. pseudotuberculosis* [21] and retains an analogous QS system [22], [23]. However the relationship between QS and regulators of the motility cascade such as *flhDC* or *fliA* may be different in *Y. pestis* when compared with *Y. pseudotuberculosis* or *Y. enterocolitica* because *Y. pestis* is non-motile because of a frame-shift mutation in the motility master regulator *flhD* [24].

There is considerable evidence to show that AHL-dependent QS plays a significant role during the biofilm mode of growth on an abiotic surface since AHL production has been detected in glass and metal surface associated biofilms produced by bacteria such as *Pseudomonas aeruginosa* [25] and *Aeromonas hydrophila* [26]. Furthermore, in a variety of bacteria, QS controls the target genes required for different stages of biofilm development from adherence and aggregation to maturation and dispersal (for review see [27]). In addition QS determines the physiological response of biofilm communities to antimicrobial agents and host defences [28], [29].

In the present paper we sought to determine whether biofilm formation by *Y. pseudotuberculosis* on a living motile surface i.e. on *C. elegans* is an interactive, QS-dependent process. The results obtained revealed that QS in *Y. pseudotuberculosis* reciprocally regulates the *C. elegans* biofilm phenotype with type III secretion *via* the major motility regulators *flhDC* and *fliA*. Consequently the induction of type III secretion attenuates biofilm formation on *C. elegans* which can be restored in a QS mutant either by curing the pYV virulence plasmid from the *ypsI/ytbI* mutant, by growing YpIII under conditions permissive for type III needle formation but not Yop secretion or by mutating the type III secretion apparatus gene, *yscJ*, a key component of the type III injectisome.

## Results

### *Y. pseudotuberculosis* produces AHLs when growing as a biofilm on the surface of *C. elegans*

**Figure 1:**
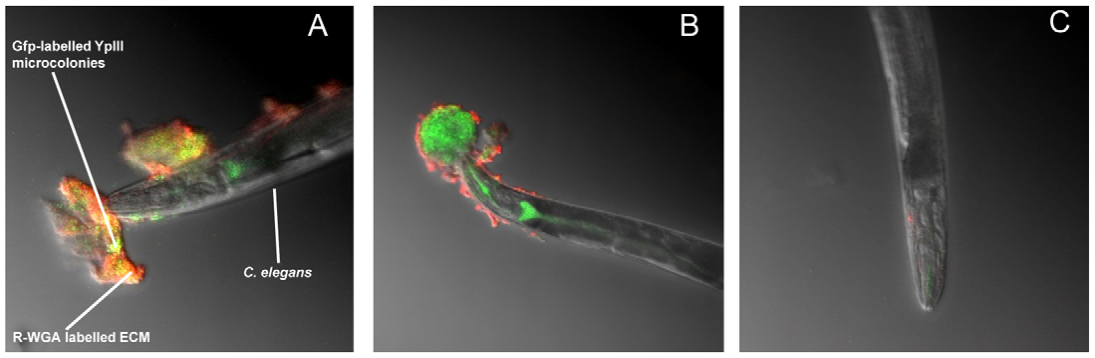
*Y. pseudotuberculosis* QS mutants are attenuated for biofilm formation on *C. elegans*. (A) Confocal image showing *C. elegans* heavily infected with *Y. pseudotuberculosis* YpIII embedded in a biofilm ECM which surrounds the anterior end of *C. elegans* and is spreading to other areas of the worm surface. Green, Gfp-labelled *Y. pseudotuberculosis* red, WGA-R binding to the ECM yellow, red and green overlay. (B) Confocal image of a *Y. pseudotuberculosis* YpIII biofilm on *C. elegans* after 48 h in which only the outer surface of the ECM stains with WGA-R which no longer penetrates deep into the biofilm. (C) *C. elegans* infected with the *Y. pseudotuberculosis ypsI/ytbI* double mutant.

**Figure 2:**
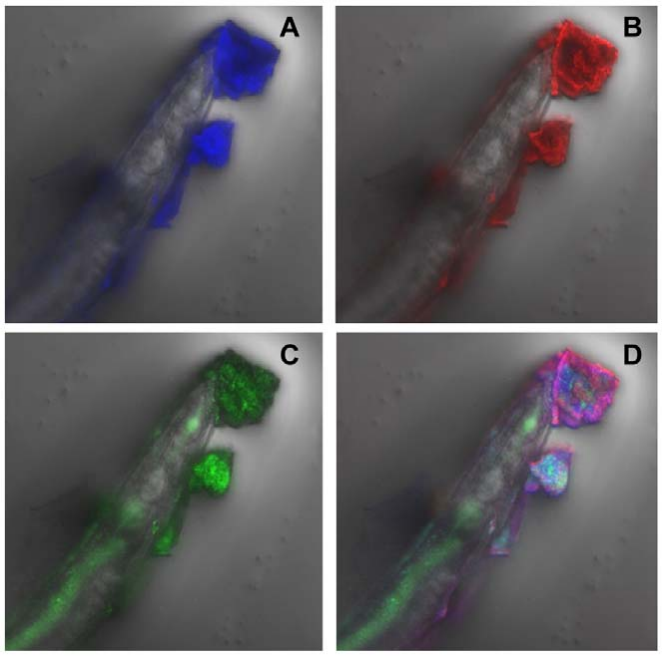
*Y. pseudotuberculosis* biofilm ECM on *C. elegans* contains extracellular DNA. (A) The ECM fluoresces blue when stained with DAPI consistent with the presence of extracellular DNA. (B), (C) and (D) show the same image labelled with WGA-R (B; red), Gfp-labelled YpIII (C; green) and an overlay image (D) of the three fluorescent labels.

When *C. elegans* is infected with *Y. pseudotuberculosis* YpIII harboring the *gfp*-plasmid pSB2020 and examined by confocal microscopy, the bacterial microcolonies fluoresce green and are embedded in an ECM which fluoresces red (yellow when both bacteria and matrix are combined) (**Figure 1A**) when labelled with WGA-R consistent with the presence of bacterially generated *N*-acetyl-D-glucosamine [12]. An orthogonal image of **Figure 1A** showing the depth of the biofilm in x and y planes can be seen in **Figure S1A**. After 48 h incubation the biofilms on *C. elegans* became highly resistant to WGA-R labelling and only stained red on the outer surface while the inner mass remained green (compare **Figure 1A** with **Figure 1B**). In common with bacterial biofilms formed on abiotic surfaces [30], the *Yersinia* biofilm on *C. elegans* also contains extracellular DNA as revealed by DAPI staining (**Figure 2A**).

**Figure 3:**
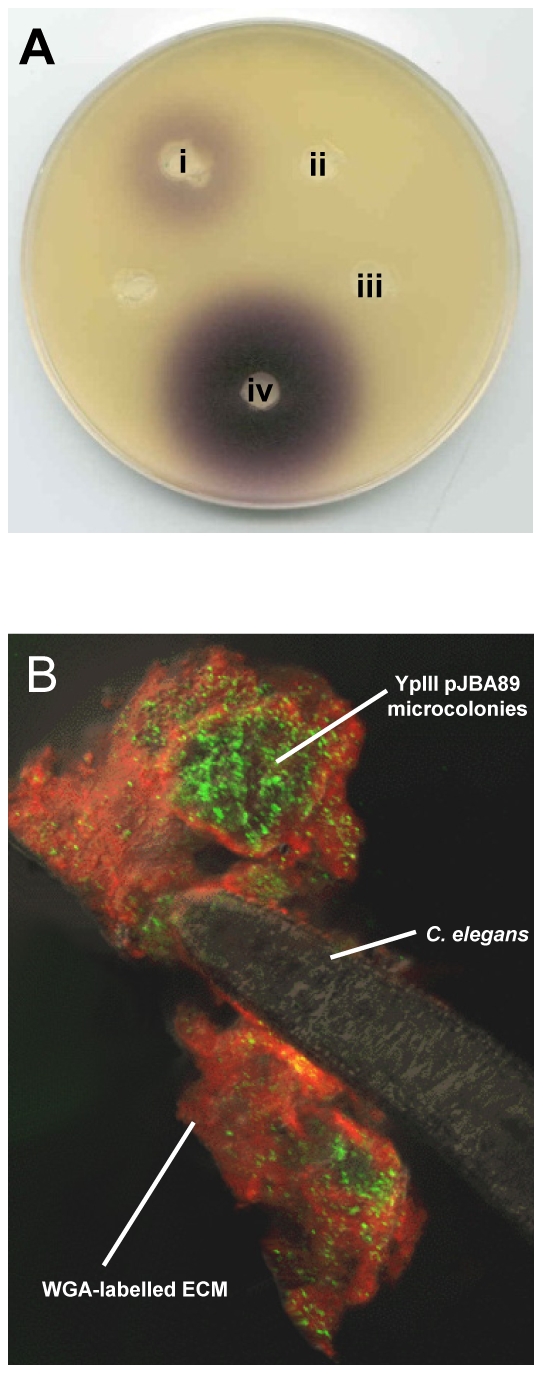
AHLs are produced in *Y. pseudotuberculosis* YpIII biofilms on *C. elegans*. (A) *C. violaceum* AHL plate assay showing that AHLs are present in a *Y. pseudotuberculosis* biofilm growing on *C. elegans*. (i) *Y. pseudotuberculosis* YpIII biofilm extract harvested from *C. elegans*; (ii) extract from nematodes grown on *E. coli* OP50 (iii) cell pellet extract from an overnight liquid culture of *Y. pseudotuberculosis* and (iv) extract of an overnight liquid culture of *Y. pseudotuberculosis* YpIII. The AHL levels collected from the biofilm appear to be present at lower levels than in the culture supernatant. (B) Confocal image showing *Y. pseudotuberculosis* YpIII transformed with the AHL reporter, pJBA89 fluorescing green in response to AHLs in the biofilm. Red and yellow represent WGA-R stain of the ECM and the overlay of red and green respectively.

To determine qualitatively whether AHLs are produced in the biofilms which accumulate on the surface of *C. elegans,* the biofilm matrix from heavily infected nematodes grown in the presence of *Y. pseudotuberculosis* for 24 h was extracted into dichloromethane and the extracts analysed using the AHL bioreporter *C. violaceum* CV026 in a well plate overlay assay [31]. As negative controls, AHL extractions were also carried out on nematodes which had been grown on *E. coli* OP50 and from the cell pellet of an overnight *Y. pseudotuberculosis* culture. Culture supernatant from the latter served as a positive control. **Figure** **3A** (**i**) shows a purple halo of violacein around the agar well which contained the concentrated nematode extract taken from worms infected with parent *Y. pseudotuberculosis*. A similar result was obtained for the positive control (**Figure** **3A** **iv**) while no violacein was observed around the negative control wells. Taken together these data indicate that AHLs are produced by *Y. pseudotuberculosis* growing as biofilms on the surface of *C. elegans*.

To confirm that AHLs are synthesised *in situ* in the biofilms, *Y. pseudotuberculosis* was transformed with the *gfp*-biosensor, pJBA89 which fluoresces green in the presence of AHLs [32]. When infected with *Y. pseudotuberculosis* pJBA89 the characteristic biofilms which form on the surface of *C. elegans* after 24 h show green fluorescent *Y. pseudotuberculosis* pJAB89 embedded in the red WGA-R labelled biofilm matrix (**Figure 3B**) which were indistinguishable from those presented **Figure 1A**.

### Quorum sensing regulates biofilm development on the surface of *C. elegans*

**Figure 4:**
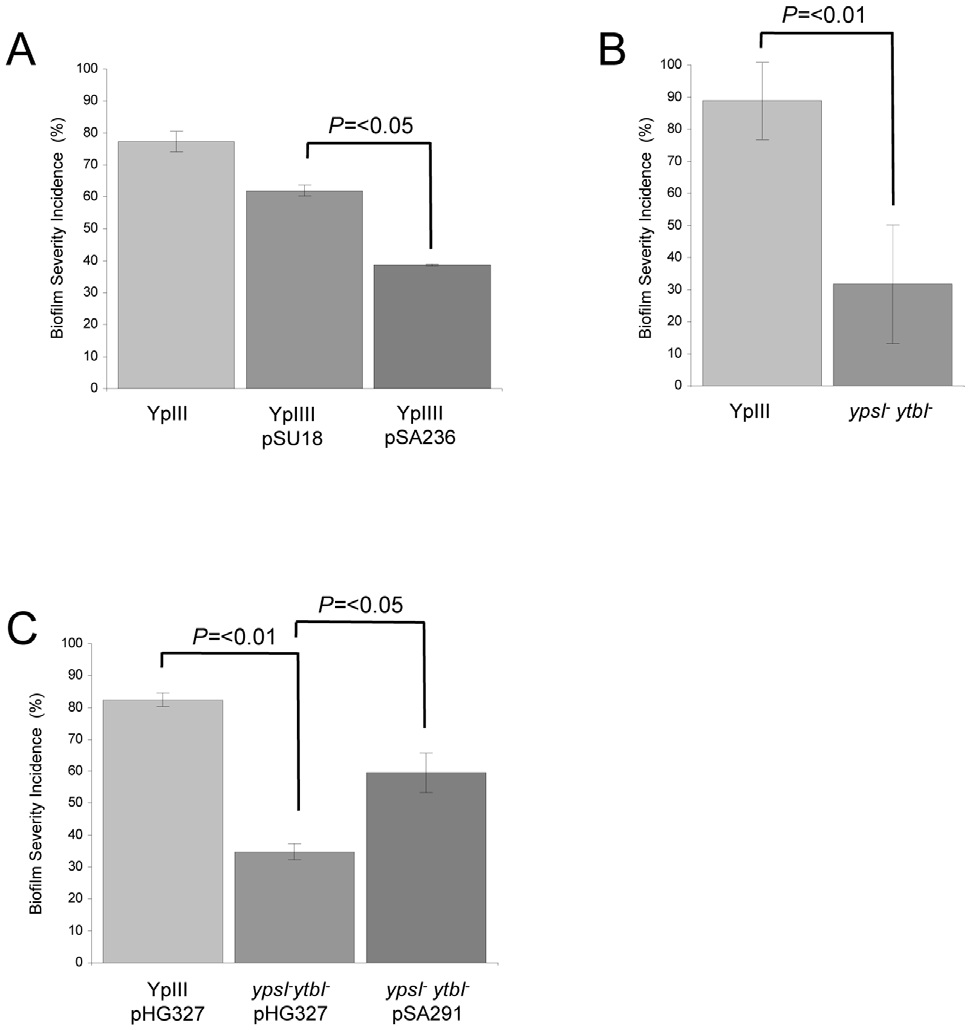
QS controls *Y. pseudotuberculosis* biofilm formation on *C. elegans.* Biofilm severity as a measurement of biofilm formation by *Y. pseudotuberculosis* YpIII, transformed with the vector pSU18 or expressing the AHL lactonase AiiA on plasmid pSA236 (A) and for the *ypsI/ytbI* mutant (B) and complemented *ypsI/ytbI* mutant (C).

Since AHLs were detected in the biofilms formed on *C. elegans*, we used two approaches to determine whether QS was required for biofilm development on the nematode surface. Firstly, we exploited the lactonase, AiiA which hydrolyses the ester bond within the AHL homoserine lactone moiety generating the corresponding, inactive, *N*-acylhomoserine compound [33]. When *aiiA* is introduced into *Y. pseudotuberculosis* on the pSU18 derivative pSA236, the AHLs produced are hydrolysed, so generating an AHL-negative phenotype [19]. By comparing the parent YpIII strain with YpIII transformed with either the pSU18 control vector or pSA236, we evaluated the contribution of AHL-dependent QS to biofilm development. For these experiments, a biofilm severity incidence was calculated for the infected *C. elegans* population after 24 h incubation. Each nematode was assigned a score between 0 and 3 related to the severity of biofilm accumulation (examples of scores 0 and 3 can be taken from the biofilms shown in **Figure 1A and C**; and scored of 1 and 2 from **Figure S1 B** and **C** respectively). These assays revealed that *Y. pseudotuberculosis* and *Y. pseudotuberculosis* pSU18 had biofilm severity indices of 77.3% and 62.0% respectively. When *C. elegans* infected *Y. pseudotuberculosis* pSU18 were compared to nematodes infected with *Y. pseudotuberculosis* pSA236 the biofilm severity incidence was reduced to 38.7% (*p* = <0.05 and *n* = 3 respectively) (**Figure 4A**).

**Figure 5:**
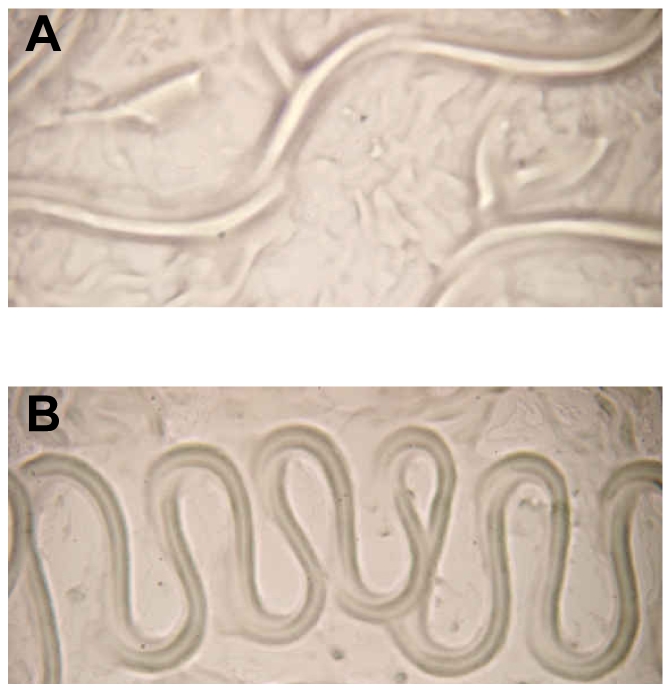
Aberrant translocation of *C. elegans* on *Y. pseudotuberculosis.* (A) *E. coli* OP50 and (B) *Y. pseudotuberculosis* YpIII. Worms infected with either the *ypsI/ytbI* or *ypsR/ytbR* mutants translocate normally and make tracks in the agar similar to those seen in (A) and only begin to show signs of aberrant movement comparable with (B), 3–4 h post infection.

Secondly we carried out *C. elegans* infection assays using *Y. pseudotuberculosis* YpIII QS mutants transformed with the constitutive *gfp*-plasmid, pSB2020. These included an AHL negative mutant in which both AHL synthase genes (*ypsI* and *ytbI)* have been disrupted and a second double mutant in which the two QS response regulators, *ypsR* and *ytbR* have been disrupted [18], [19]. When compared with the parent *Y. pseudotuberculosis* YpIII strain (**Figure 1A**), biofilm development was severely delayed in the *ypsI/ytbI* double mutant formed little or no biofilm (compare **Figure 1A and 1C**). Similar results were obtained for the *ypsR/ytbR* double mutant (data not shown). In addition, nematodes grown on YpIII, in contrast to those grown on *E. coli* OP50, exhibit exaggerated body bends (**Figure 5**), are unable translocate within 1.5 h and by 5 h become moribund. In contrast, *C. elegans* infected with either the *ypsI/ytbI* mutant or the *ypsR/ytbR* mutant translocate normally and make tracks in the agar which are identical to those presented in **Figure 5A** and only began to show signs of aberrant movement 3–4 h post infection. After 96 h growth, both the *ypsI/ytbI* and *ypsR/ytbR* mutants formed severe biofilms on the nematodes. In addition, we calculated a biofilm severity incidence for each yersinia strain. **Figure 4B** shows that after 24 h there is an ∼3 fold reduction in the amount of biofilm on nematodes infected by the *ypsI/ytbI* double mutant compared with the parent (32% compared with 89%; *p* = <0.01 *n* = 4). Similar results were obtained for the *ypsR/ytbR* double mutant (data not shown). Genetic complementation of the *ypsI/ytbI* mutation with pSA291 (**Figure 4C**) partially restored the biofilm severity incidence to that of the parent strain (Parent pHG327 (82%) compared with the *ypsI/ytbI* mutant pHG327 (35%) (*p* = 0.001 *n* = 3) and *ypsI/ytbI* mutant pSA291 (60%) compared with *ypsI/ytbI* mutant pHG327 (35%) (*p* = <0.05 *n = 3*)).

These data demonstrate that the loss of AHL synthesis either via enzyme-mediated inactivation or by mutagenesis of the AHL synthases results in the attenuation of biofilm formation on *C. elegans*. Consequently QS is pivotal to the timing and severity of biofilm development on *C. elegans*.

### Flagellar-mediated motility is not required for biofilm development on *C. elegans*

**Figure 6:**
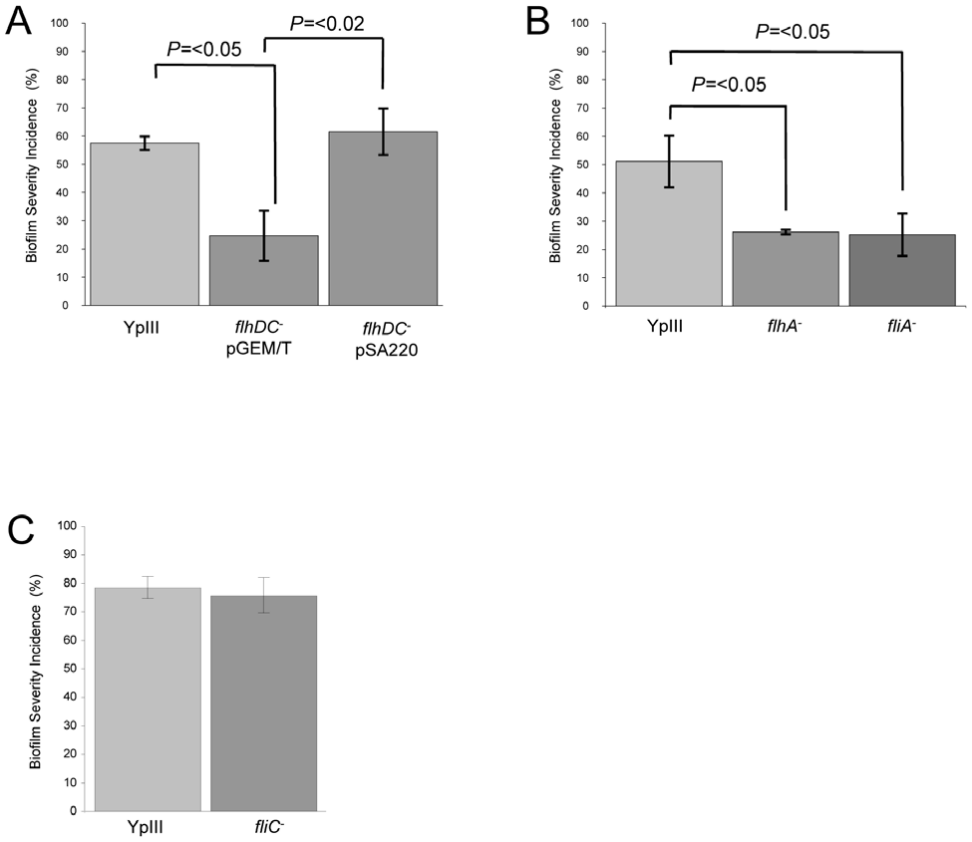
*Y. pseudotuberculosis* strains with mutations in *flhDC, fliA or flhA* but not *fliC are* attenuated for biofilm formation. Biofilm severity indices are shown for *flhDC* and the complemented *flhDC* mutant (A), *flhA* and *fliA* (B) and *fliC* (C).

Since the *ypsR/ypsI* and *ytbR/ytbI* loci are both involved in the regulation of motility via *flhDC* and *fliA* which code for the motility master regulator and flagellar specific sigma factor respectively [19], we sought to determine whether these downstream regulators contribute to the *Yersinia/C. elegans* biofilm phenotype. **Figure 6A** shows that the *flhDC* mutant was impaired in its ability to form biofilms on the surface of *C. elegans* (biofilm severity incidence for the parent of 57.5% compared to 24.7% for the *flhDC* mutant (*p* = <0.05, *n* = 3)) and genetic complementation of *flhDC* using pSA220 increased the biofilm severity to 61.6% when compared with the *flhDC* mutant (*p* = <0.02, *n* = 3). **Figure 6B** shows that the biofilm severity incidence for the *fliA* mutant was also reduced when compared with the parent (*p* = <0.05, *n* = 3). Since both regulators control swimming motility and as *flhDC* and *fliA* mutants are non-motile, these data suggested that biofilm formation may depend on flagellar-mediated motility. To explore this possibility, we first constructed a flagellin-negative strain by mutating the flagellin structural gene, *fliC.* This non-motile mutant formed biofilms on nematodes which were indistinguishable from the parent *Y. pseudotuberculosis* strain (**Figure 6C** and data not shown). Consequently, flagellar-mediated motility is not a necessary pre-requisite for biofilm formation on *C. elegans*. However, in *Y. enterocolitica*, the flagellar type III secretion apparatus may also secrete non-flagellar proteins termed ‘Fops’ (for Flagellar outer proteins) such as the phospholipase, YplA [34]. Since flagellin structural mutants still secrete Fops, we constructed a *flhA* mutant since this gene codes for a structural component of the flagellar protein export apparatus [35] and *flhA* mutants have been reported not to secrete Fops [34]. In common with the *flhDC* and *fliA* mutants and when compared to the parent, the *flhA* mutant exhibited attenuated biofilm formation (**Figure 6B**) (*p* = <0.05, *n* = 3), a finding which implies a possible role for a secreted protein(s).

**Figure 7:**
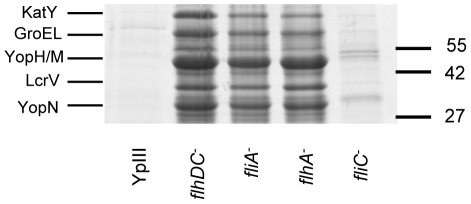
SDS-PAGE protein profiles of cell free supernatants prepared from *Y. pseudotuberculosis* YpIII parent, *flhDC*, *fliA*, *flhA* and *fliC* mutants grown at 30°C. The up-regulated proteins YopN, YopM/H, LcrV, KatY and GroEL were identified by MALDI-TOF MS. Molecular masses of the marker proteins are in kDa.

To determine whether any secreted proteins could be involved in biofilm development on *C. elegans,* we first examined the extracellular protein profiles of the *Y. pseudotuberculosis flhDC*, *fliA*, *flhA* and *fliC* mutants grown overnight in LB_mops_ at 30°C. **Figure 7** shows that compared with the parent strain and *fliC* mutant, numerous proteins are up-regulated in each of the other motility mutants. MALDI-TOF MS analysis identified three of the major protein bands as YopM/H (41/51 kDa, these two proteins often co-migrate and could not be distinguished by MALDITOF sequencing), LcrV (37 kDa) and YopN (32 kDa) all of which are encoded on the pYV virulence plasmid and secreted by the Ysc-Yop type III secretion system. Two further up-regulated proteins were identified as KatY and GroEL which are not related to the Yop virulon (**Figure 7**).

### Quorum sensing represses type III secretion in *Y. pseudotuberculosis*

**Figure 8:**
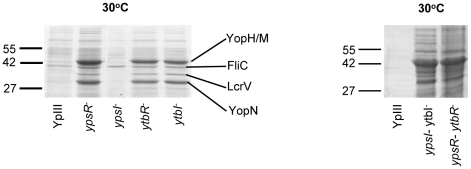
SDS-PAGE protein profiles of the *Y. pseudotuberculosis* parent and the QS mutants prepared from cell-free supernatants grown at 30°C. YopN, YopH/M, LcrV, and FliC were identified by MALDI-TOF MS. Molecular masses of the marker proteins are in kDa.

In contrast to the YpIII parent strain which only secretes Yops at 37°C in the absence of Ca^2+^ both *flhDC* and *fliA* mutants clearly secrete Yops at 30°C in the presence of Ca^2+^. Since both of these motility regulators are controlled by QS in *Y. pseudotuberculosis* [19], these data suggested that elements of the Yop virulon are also likely to be QS-controlled. **Figure 8** shows that when grown in LB_Mops_ at 30°C overnight, at least 4 extracellular proteins are up-regulated in the *ypsI/ytbI* and *ypsR/ytbR* double mutants compared with the parent strain. The same proteins are also up-regulated in the *ypsR*, *ytbR* and *ytbI* single mutants whereas the *ypsI* mutant exhibits the same profile as the parent strain. MALDI-TOF MS analysis identified the proteins as YopM/YopH, FliC, LcrV and YopN. These proteins were also present in supernatants from the same mutants after growth at 37°C in LB_Mops_ but absent from the parent and *ypsI* mutant (**Figure S2**). In contrast, Yop proteins were absent from the supernatants of all of the strains grown at 22°C although two proteins, the flagellar capping protein (FliD; 48.6 KDa) and flagellin (FliC; 45 KDa) were up-regulated (data not shown).

### The pYV plasmid inhibits biofilm formation by the *Y. pseudotuberculosis ypsI/ytbI* and *flhDC* mutants

**Figure 9:**
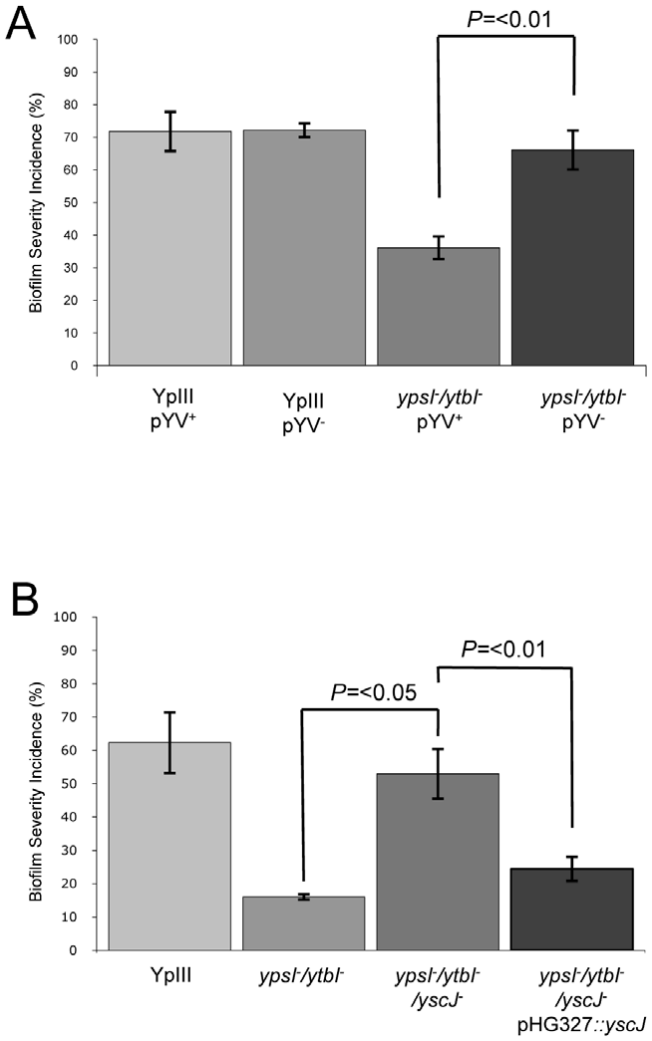
Impact of pYV and type III secretion on biofilm formation by *Y. pseudotuberculosis* YpIII on *C. elegans*. Biofilm severity indices are shown for (A) YpIII and the *ypsI/ytbI* mutant with or without pYV and (B) YpIII and the *ypsI/ytbI* mutant compared with the *ypsI/ytbI/yscJ* triple mutant and the triple mutant complemented with a plasmid borne copy of *yscJ*.

The attenuation of biofilm formation on *C. elegans* observed for both the motility and QS mutants in conjunction with the elevated secretion of Yop virulon proteins at non-permissive temperatures raised the possibility that induction of type III secretion blocks biofilm development. Consequently, we predicted that biofilm formation would be restored in *Y. pseudotuberculosis ypsI/ytbI* and *flhDC* mutants cured of the pYV plasmid. To explore this hypothesis, we cured the pYV plasmid from the parent, *ypsI/ytbI* and *flhDC* mutants by repeated selection on CRMOX agar plates. The presence or absence of the pYV plasmid had no effect on the ability of the *Y. pseudotuberculosis* YpIII parent strain to form a biofilm on *C. elegans* (**Figure 9A**). However when similar experiments were performed using the *ypsI/ytbI* double mutant (**Figure 9A** and compare with **Figure 4B**) or *flhDC* (data not shown) cured of pYV, biofilm formation on *C. elegans* was restored to parental strain levels when compared with the biofilm levels observed on the *ypsI/ytbI* pYV^+^ double mutant (*p* = <0.01, *n* = 3). These data suggest that under these conditions, AHL-mediated QS represses the expression of a pYV gene(s) which would otherwise prevent biofilm formation.

To gain further evidence in support of a biofilm inhibitory role for pYV Yop virulon component(s), *C. elegans* was infected with the parent *Y. pseudotuberculosis* grown in LB_mops_ MOX, conditions which promote Yop secretion (i.e. 37°C in the absence of Ca^2+^) rather than in LB_mops_ at 30°C in which Yops will not be secreted. These seed cultures were then transferred to NGM plates containing MgCl_2_ and sodium oxalate to chelate Ca^2+^. Under such pre-conditions, the type III system is induced and no biofilms were formed on *C. elegans* (data not shown) providing additional support that induction of the Yop virulon prevents biofilm formation on *C. elegans*.

### Biofilm formation on *C. elegans* is inhibited by induction of the type III injectisome

To demonstrate unequivocally that the inhibition of biofilm formation on *C. elegans* observed for the *Y. pseudotuberculosis ypsI/ytbI* mutant depends on the induction of functional type III secretion system rather than other genes present on the pYV plasmid, we modified the *ypsI/ytbI* mutant by mutating *yscJ*. This gene codes for a key component of the Ysc injectisome required for the assembly of a functional type III secretion apparatus [36]. Cell free culture supernatants taken from the *ypsI/ytbI/yscJ* triple mutant grown in LB_Mops_ at 30°C were examined by SDS-PAGE. This confirmed that, in contrast to the *ypsI/ytbI* mutant, Yop proteins were no longer secreted (data not shown). Yop secretion in the triple mutant grown under these conditions could however be restored by complementation with a plasmid-borne copy of *yscJ* (pHG::*yscJ*; data not shown). In the *C. elegans* biofilm assays, the biofilm severity index of the *ypsI/ytbI/yscJ* triple mutant was ∼4-fold higher than that of the *ypsI/ytb* double mutant (*p* = <0.05, *n* = 3) and comparable with that of the parent strain (**Figure 9B**). When the triple mutant was compared to its complemented counterpart containing a functional copy of *yscJ* (on plasmid pHG::*yscJ*) biofilm severity was reduced ∼two-fold (*p* = <0.01, *n* = 3) back to levels comparable with the *ypsI/ytbI* double mutant (**Figure 9B**). These results are consistent with a role for the type III injectisome in preventing biofilm development on *C. elegans* and demonstrate that either the type III needle or the secreted Yop proteins or both prevent biofilm development on *C. elegans*. To attempt to differentiate between these three possibilities, we grew the *Y. pseudotuberculosis* YpIII parent strain at 37°C in the presence of calcium which results in type III needle assembly but not Yop secretion [37]. This is because Ca^2+^ prevents Yop effector secretion even in the presence of a fully formed injectisome. YpIII was then subcultured onto NGM medium supplemented with calcium. When pre-cultured under these conditions and used to infect *C. elegans* at 22°C, *Y. pseudotuberculosis* YpIII failed to form a biofilm on *C. elegans*. The infected worms were indistinguishable from that shown in **Figure 1C** suggesting that the type III needle rather than the Yop effectors was responsible for preventing biofilm development.

## Discussion

On abiotic surfaces, bacterial biofilm formation is generally considered as a step-wise process initiating from individual cells adhering to a substratum leading to microcolony formation, biofilm maturation and finally dispersal to new sites [38]–[42]. Although the nature and development of biofilms formed on biotic surfaces have not been as thoroughly investigated, biofilm development by *Y. pseudotuberculosis* on *C. elegans* involves attachment and maturation stages and the ECM contains both carbohydrate and extracellular DNA. Whether the DNA present in the biofilm is bacterial or nematode-derived has yet to be established. However, the WGA-stained carbohydrate present in the ECM appears to be bacterially-derived since it is present in the lawns of *Y. pseudotuberculosis* prior to the addition of nematodes which are not labelled by WGA [43]. The WGA-stained ECM carbohydrate could be either peptidoglycan which contains *N*-acetyl glucosamine in the sugar backbone [44] or polymeric *N*-acetyl-D-glucosamine or both. *Y. pestis* strains with mutations in the *hmsHFRS* locus, which is responsible for the biosynthesis of a poly β-1,6-*N*-acetyl-D-glucosamine-like polysaccharide [45], are defective for biofilm accumulation on *C. elegans* implying that this exopolysaccharide plays an essential role. An intact *hmsHFRS* is also required for biofilm formation on *C. elegans* by both *Y. pseudotuberculosis* and *Xenorhabdus nematophila* [46].

Apart from the *hmsHFRS genes*, other yersinia genes currently known to be required for biofilm formation on *C. elegans* include two genes involved in LPS biosynthesis, two genes of unknown function and a potential hybrid two component regulatory protein [6]. Both RcsA (a phosphorelay accessory protein which functions in concert with the response regulator, RcsB) and PhoP negatively regulate the formation of *Y. pseudotuberculosis* biofilms on nematodes [47] while the action of PhoP appears to be mediated at least in part by the down-regulation of HmsT [48]. This is interesting since HmsT is a cyclic diguanylate (c-di-GMP) synthase and c-di-GMP metabolism plays an important role in biofilm formation in many different bacteria including *Y. pestis* [49], [50].

Depending on the organism, QS may be involved in the early attachment or later maturation stages of biofilm development on abiotic surfaces [27]. In pathogens such as *P. aeruginosa*, QS is responsible for controlling the expression of key components of the biofilm extracellular matrix including exopolysaccharides and extracellular DNA release as well as the refractory nature of biofilms to host defences and antimicrobials [27]. The contribution of QS to yersinia biofilm development on *C. elegans* has not previously been investigated although for *Y. pseudotuberculosis*, QS controls cell aggregation (a type of suspended biofilm) in liquid culture [18]. A *Y. pestis* strain with combined mutations in *ypsR/ypsI, ytbR/ytbI* and *luxS* formed a similar biofilm on glass cover slips to the parental strain which could not be distinguished by crystal violet or Congo red staining although a very mild defect was observed using confocal microscopy [51]. Here, for *Y. pseudotuberculosis* YpIII, we have shown that AHL-dependent QS is functional in biofilms formed on *C. elegans* by demonstrating (i) the presence of AHL signal molecules within the nematode-associated biofilm matrix and (ii) that YpIII strains in which AHL biosynthesis is abrogated either by expressing an AHL-inactivating enzyme *in situ* or by mutating the AHL synthases (YpsI and YtbI) are attenuated for biofilm formation. Because *Y. pseudotuberculosis* YpIII does not form biofilms on polystyrene surfaces [6], these data indicate that the QS-dependent pathway for biofilm formation on *C. elegans* is different from that on abiotic surfaces. While QS signals have previously been identified in pseudomonas and aeromonas biofilms on abiotic surfaces [25], [26] to our knowledge they have not previously been detected directly in biofilms growing on a living, biotic surface. AHLs have however been shown to be produced in the tissues of mice infected with *Y. enterocolitica* [52] although no evidence was presented for biofilm formation in this acute experimental infection model.

In *Y. pseudotuberculosis*, YpsRI and YtbRI form a QS hierarchy in which *ypsR* is auto-regulated and also controls the expression of *ypsI*, *ytbI* and *ytbR*; YtbR also regulates *ytbI* expression [19]. In common with the *ypsI/ytbI* double synthase mutant, the *ypsR/ytbR* double response regulator mutant was also attenuated for biofilm development on *C. elegans*. The *ypsR/ytbR* mutant however produces a similar AHL profile to that of the parent strain [19] and therefore AHL production *per se* is not required for biofilm formation. The intermediate level biofilms formed by the single *ypsR*, *ytbR* and *ytbI* mutants (data not shown) reflect the interdependent nature of the *Y. pseudotuberculosis* QS system while the lack of biofilm attenuation observed for the *ypsI* mutant suggested that the AHLs synthesized via YtbI are primarily responsible for the biofilm phenotype observed.

A number of Gram-negative bacterial species rely on flagellar-mediated motility for specific stages of biofilm formation [38]. For example, in *E. coli*, mutations which lead to either the loss of flagella or flagella function (which include *fliC* or *flhD*) are unable to form mature biofilms indicating that the presence of functional flagella is a pre-requisite for biofilm development in a PVC attachment model [53]. Similarly, non-motile yet flagellate *P. aeruginosa* PA01 *flgK* mutants and *Erwinia carotovora fliC* or *motA* mutants cannot form biofilms on PVC surfaces [54], [55]. Furthermore, in *Y. enterocolitica*, mutations that abolish the structure or rotation of the flagellar greatly reduced biofilm formation in PVC microplate assays [56]. Thus, given the links between biofilm formation, flagella-mediated motility and the regulation of the two key motility regulators, *flhDC* and *fliA* by QS in *Y. pseudotuberculosis* [19], we investigated the contribution of motility to biofilm formation on *C. elegans.* Surprisingly, a *Y. pseudotuberculosis fliC* mutant formed similar biofilms to the parent strain indicating that on the nematode, the presence of flagellar is not a pre-requisite for biofilm formation. This provides further evidence to suggest that the biofilm developmental pathway on the living nematode surface is distinct from that occurring on an abiotic surface. Since flagellins are potent inducers of the innate immune response and are often considered as flags revealing the presence of bacteria [57], it may therefore be advantageous for *Yersinia* to repress their expression during growth on living surfaces.

Despite the lack of biofilm attenuation for the *fliC* mutant, non-motile strains with mutations in *flhA*, a structural component of the flagellar export apparatus as well as the motility cascade regulators, *flhDC* and *fliA* were significantly attenuated. Since QS governs the expression of key motility regulators [19] these data suggested that biofilm formation on *C. elegans* by *Y. pseudotuberculosis* was linked to QS via the motility cascade. As *Y. pestis* has a frameshift mutation in *flhD,* biofilm formation on *C. elegans in Y. pestis* may well be governed differently to *Y. pseudotuberculosis*.

*Y. enterocolitica* secretes FOP proteins such as YplA via the flagellar type III secretion apparatus [34]. Consequently, we considered it possible that the loss of *Y. pseudotuberculosis* FOP proteins by mutation of the motility genes may have been responsible for biofilm attenuation. However, SDS-PAGE analysis of the extracellular protein profile of these strains did not reveal any novel FOP proteins but rather the presence of several proteins associated with the Yop virulon and type III secretion. In particular, LcrV which is associated with the tip of the injectisome and with pore formation across the host cell membrane, YopN, a plug considered to limit Yop effector translocation through the needle and YopH, a phosphotyrosine phosphatase effector protein which inhibits phagocytosis (reviewed by [2]). Our findings are consistent with observations made by [58] that deletion of *flhDC* resulted in the up-regulation of the *yop* regulon in *Y. enterocolitica* as a consequence of FlhDC-mediated repression of the Yop virulon regulator gene, *virF*.

Since QS in *Y. pseudotuberculosis* regulates *flhDC* and *fliA* [19] we also examined cell free supernatants of strains with mutations in the *ypsRI* and *ytbRI* loci for the up-regulation of Yop virulon proteins. Apart from the single *ypsI* mutant, which exhibited the parental phenotype, each of the QS mutants exhibited the same protein profile on SDS-PAGE as the *flhDC* and *fliA* mutants when grown at 30°C in the presence of Ca^2+^. Since both injectisome and Yop effector proteins were up-regulated, these data suggest that QS represses the Yop virulon via the actions of FlhDC on *virF*. In addition, it is clear that mutation of QS results in the loss of both the temperature and Ca^2+^ dependence characteristic of type III secretion in *Yersinia*. Thus in *Y. pseudotuberculosis*, QS positively regulates motility but negatively controls type III secretion indicating that both phenotypes are population dependent. This would suggest that in the planktonic phase at high population densities in the presence of eukaryotic target cells, Yop secretion would be shut down in favour of bacterial migration to new sites where a fall in QS signal concentrations would stimulate the resumption of Yop secretion.

With respect to the biofilm phenotype of the QS and motility mutants, the de-repression of type III secretion at temperatures below 37°C suggested that type III secretion blocked biofilm formation on *C. elegans*. Since the Yop virulon genes are located entirely on the pYV plasmid, we examined the biofilm phenotype of the plasmid-cured parent, *ypsI/ytbI* and *flhDC* mutants respectively. The loss of pYV from the parent *Y. pseudotuberculosis* strain had no impact on biofilm formation an observation which is fully in agreement with Joshua *et al*., (2003) [6] who examined both YpIII and a range of *Y. pseudotuberculosis* strains with or without the virulence plasmid. However the attenuation of biofilm formation observed for both the *ypsI/ytbI* and *flhDC* mutants could be overcome by curing pYV, a finding which implied that QS represses the expression of pYV encoded gene(s) which block biofilm formation in the presence of Ca^2+^ and at 22°C, the temperature at which the *C. elegans* assays are carried out. Additional support for these observations was obtained when seed cultures of the *Y. pseudotuberculosis* parent strain were grown under conditions permissive for Yop release (37°C in the absence of Ca^2+^) and then transferred onto Ca^2+^-free modified NGM plates at 22°C whereupon biofilms did not form on *C. elegans*.

To rule out the possibility that other genes located on the pYV plasmid were responsible for the biofilm phenotype rather than the presence of a functional type III secretion system, we introduced a *yscJ* mutation into the *ypsI/ytbI* double mutant. The newly generated triple mutant resulted in the loss of type III secretion at 30°C in the presence of Ca^2+^ and the restoration of biofilm formation on *C. elegans*. This strongly implies that the presence of an intact injectisome blocks biofilm formation on *C. elegans*. However, these data alone could not determine whether the reduction in biofilm was due to the presence of an intact injectisome, extracellular Yops or both. Evidence to suggest that the type III injectisome rather than the Yop effectors were responsible for attenuating biofilm formation on *C. elegans* was obtained by first conditioning seed cultures of *Y. pseudotuberculosis* at 37°C in Ca^2+^ containing media prior to carrying out biofilm assays. We reasoned that the conditioned *Y. pseudotuberculosis* cells would possess intact injectisomes but would not release Yops [59]–[63]. Furthermore, the presence of Ca^2+^ in the NGM agar would continue to suppress Yop secretion during the biofilm assays. When biofilm assays were performed using pre-conditioned *Y. pseudotuberculosis* cells biofilm formation was suppressed. These data appear to preclude a requirement for extracellular Yops in order for biofilm formation to take place. The simplest explanation is that the presence of the fully formed needle acts as a physical barrier which blocks the interaction between a key, chromosomally encoded bacterial surface component and the nematode surface. This would also be consistent with the loss of biofilm formation which results from the mutation of a number of *C. elegans* surface-determining genes [64], [65]. However, at this stage we cannot rule out the possibility that contact between the injectisome and *C. elegans* results in the repression of as yet unidentified genes required for biofilm formation.

## Materials and Methods

### Strains and growth conditions

The *Y. pseudotuberculosis*, *Escherichia coli* and *C. elegans* strains and the plasmids used in this study are listed in **Table S1** and **Table S2** respectively. To aid visualisation of *Y. pseudotuberculosis* in biofilm assays, the bacterial cells were transformed with pSB2020 [66] which constitutively expresses *gfp3.* To determine whether biofilm formation on *C. elegans* could be attenuated by AHL hydrolysis, *Y. pseudotuberculosis* YpIII was also transformed with the lactonase gene, *aiiA* on pSA236 as described before [19]. Except where stated, bacterial cultures were routinely grown with shaking at 200 rpm in L broth Lennox [67] or on agar plates containing the appropriate antibiotics buffered to pH 6.8 with Mops (3-*N*-morpholino) propanesulphonic acid (YLB_mops_) to reduce alkaline hydrolysis of AHLs during bacterial growth [68]. To promote *yop* expression at 37°C some experiments were performed in YLB_mops_ supplemented with MgCl_2_ (20 mM) and sodium oxalate (20 mM) as previously described [58]. Where required, pYV was cured from *Y. pseudotuberculosis* by the repeated sub-culture of white colonies onto Congo red-magnesium oxalate (CRMOX) plates [69].

The *C. elegans* wild-type (N2 Bristol) strain was obtained from the *Caenorhabditis* Genetics Centre (University of Minnesota, St. Paul, MN) and maintained on modified NGM plates [70] lacking MgCl_2_, seeded with *E. coli* OP50 unless otherwise stated. For Yop induction assays NGM was supplemented with MgCl_2_ (20 mM) and sodium oxalate (20 mM) but CaCl_2_ was omitted.

### *Y. pseudotuberculosis*/*C. elegans* biofilm assay

NGM plates were seeded with 1 ml of the appropriate *Y. pseudotuberculosis* strain grown overnight at 30°C unless otherwise stated. For some *C. elegans* biofilm experiments, NGM agar plates were modified by the addition of sodium oxalate (20 mM) and MgCl_2_ (20 mM) to promote Yop secretion. For the assays in which biofilm severity incidence was calculated, *Y. pseudotuberculosis* were spread evenly over the agar surface, dried to remove excess liquid and 20–30 young adult *C. elegans* were aseptically transferred to the seeded plates. After incubation for 22°C for 24 h (unless otherwise stated), the worms were examined under low magnification using a Nikon SMZ1000 microscope and biofilm accumulation was classed as level 0 if no biofilm formed (e.g. **Figure 1C**); level 1 indicating a small accumulation of biofilm around the anterior end of the worm (e.g. **Figure S1B**); level 2 denoted larger accumulations of biofilm around the anterior end of the worm with some pockets of biofilm spreading back from the head (e.g. **Figure S1C**); level 3 by large accumulations of biofilm around the anterior end of the worm which extended to other parts of the nematode body surface (e.g. **Figures 1A** **and** **2B**). Confocal images of *C. elegans* were taken using a Zeiss LSM700 inverted microscope. Replicate Z-stacks were taken at 5 µm intervals. The Zeiss Zen software package was used for image analysis. The level of biofilm accumulation on *C. elegans* was denoted as the biofilm severity incidence and was calculated according to the method of Tarr [71]: Biofilm severity incidence  =  {[∑(level X number of samples in this level)]/(highest level X total sample numbers)} X 100%. All assays in which the level of biofilm severity was assessed were carried out double blind, with at least three or four replicates and each experiment was performed more than once. The error bars shown on figures 4, 6 and 9 represent the standard deviation from the mean and when necessary independent two-sample t-tests were performed with values for *p* and *n* given in the text and on histograms where appropriate. For some experiments the presence of the *N*-acetyl-D-glucosamine in the ECM of *Y. pseudotuberculosis* biofilms was demonstrated using a wheat germ agglutinin (WGA)-rhodamine (WGA-R) conjugate as described by [12]. Extracellular DNA present in the biofilms was stained with DAPI following the method of Vilain *et al.,* [72] in which low concentrations of DAPI are demonstrated to label the extracellular biofilm matrix without penetrating the bacterial cell and staining the intracellular DNA.

To determine whether biofilm formation was attenuated when worms were infected with *Y. pseudotuberculosis* containing the AHL lactonase AiiA, *aiiA* was excised from pSA302 [19] as an *EcoR*I fragment and then sub-cloned into the chloramphenicol resistant vector pSU18 [73] to give pSA236 which was transformed into *Y. pseudotuberculosis*. pSU18 was transformed into *Y. pseudotuberculosis* to act as a vector control.

### DNA manipulations

Plasmids were isolated using the Promega Wizard system, agarose gel electrophoresis and standard methods for the preparation of competent cells, DNA ligation and electroporation were performed as previously described [20]. For the purification of DNA fragments from agarose gels, Qiaquick DNA purification columns were used (Qiagen Ltd). Restriction endonucleases, DNA ligase and other DNA modification enzymes were used according to the manufacturers' instructions (Promega).

### AHL detection

*C. elegans* infected with *Y. pseudotuberculosis* were removed from 40 NGM plates in M9 wash solution [74]. The worms were washed, the pellet extracted into dichloromethane, reconstituted into 20 µl of acetonitrile and analysed using a well plate overlay assay using the *C. violaceum* CV026 biosensor which reports the presence of AHLs by producing the purple pigment violacein [31]. To detect AHLs produced *in situ* in biofilms on the surface of *C. elegans, Y. pseudotuberculosis* and the isogenic *ypsI/ytbI* double mutant were each transformed with the AHL biosensor, pJBA89 [32] which expresses *gfp* in the presence of AHLs. Infected worms were examined using fluorescent microscopy for the presence of green fluorescent bacteria within the biofilm matrix.

### Construction of *Y. pseudotuberculosis* mutants

*Y. pseudotuberculosis* YpIII strains with deletions in *fliA, flhA, fliC* and *yscJ* were constructed as follows. The *fliA* mutant was constructed using a modified method of [75]. The primers used for mutant construction are listed in **Table S3**. Briefly, primer pairs fliA1up-F/fliA1up-R and fliA1down-F/fliA1down-R were used to amplify 510 and 511bp fragments of the up- and downstream regions of *fliA* (positions 2069236 to 2069746 and 2070363 to 2070874 in the published *Y. pseudotuberculosis* IP 32953 genome sequence [76]). Primer fliA1up-R and fliAdown-F also contained 25 and 22 bp respectively of sequence homologous to the first 25 bp and last 22 bp of kanamycin from pUC4K [77]. The kanamycin cassette was amplified from pUC4K (Pharmacia) using primer km-F and km-R under the following PCR conditions: 95°C for 5 min followed by 30 cycles of 95°C for 30 s, 56°C for 30 s and 74°C for 1 min and ending with 74°C for 5 min. The second and third step PCR conditions were as follows: 95°C for 5 min followed by 30 cycles of 95°C for 30 s, 60°C for 30 s and 74°C for 2 min and ending with 74°C for 5 min. The strategy for constructing the *flhA* and *yscJ* mutants was similar to that of *fliA*. For *flhA*, primer pairs flhA1up-F/flhA1up-R and flhA1down-F/flhA1down-R were used to amplify the up- and downstream regions of *flhA* (positions 2017164 to 2017699 and 2019587 to 2020179 on the published IP 32953 *Y. pseudotuberculosis* genome sequence) whereas for *yscJ*, primer pairs YscJaFor/YscJupR-Tet and YscJdownF-tet/YscJbRev were used to amplify the up- and downstream regions of *yscJ* (positions 59172 to 59743 and 60344 to 61135) on the published *Y. pseudotuberculosis* IP 32953 pYV virulence plasmid sequence. For *flhA,* primer flhA1up-R and flhA1down-F each contained 19 bp of sequence homologous to the first 19 bp or last 19 bp of the kanamycin cassette from pUC4K whereas for *yscJ*, YscJupR-Tet and YscJdownF-tet contained 21 bp or 22 bp of sequence homologous to a tetracycline cassette which was amplified as a 1191 bp product from pBlue-tet (a source of the tetracycline cassette initially amplified from pBR322 using primers Tet1 and Tet2 [19] and cloned into pBluescript as an *xhoI* fragment). All PCR conditions were the same as those for the construction of the *fliA* mutant.

To complement yscJ, primers YscJF-XbaI and YscJR-SalI were used to amplify an 842 bp product from Y. pseudotuberculosis (positions 59686 to 59703 on the IP32953 published sequence) which, after cloning into pBluescript and sequencing was excised as a KpnI and PstI fragment and sub-cloned into the low copy number vector pHG327 [78]. The resulting plasmid, pHG::yscJ was transformed into the Y. pseudotuberculosis ypsI/ytbI double mutant.

Colony PCR was used to amplify a *fliC* homologue from *Y. pseudotuberculosis* using the primers DC1 and DC2 and cloned into pGEMT/easy (Promega) to give pfliC. Sequencing revealed the 1,515 bp fragment to have an open reading frame of 1,110 bp and predicted protein product of 396 amino acids that shared significant amino acid similarity to several FliC homologues and was subsequently termed fliCYp (Genbank accession number AY244555). To construct a *fliC* mutant 616 bp was removed from pfliC using *Csp*45I and replaced with a kanamycin cassette from pUC4K (Pharmacia) as a blunt end fragment. The resulting construct was cloned into pDM4 as a *Sph*I-*Spe*I fragment (pDM *fliC*-Km) and stably integrated into the chromosome of *Y. pseudotuberculosis* as previously described [18], [19].

To complement the *Y. pseudotuberculosis* YpIII *flhDC* mutant [19] *flhDC* was amplified by PCR (primers FlhD_F_ and FlhC_R_), cloned into pGEMT/Easy (Promega) and the resulting pGEM::*flhDC* construct, pSA220 was transformed into the *Y. pseudotuberculosis flhDC* mutant. The *flhDC, flhA, fliA* and *fliC* mutants were examined for motility using swim agar plate assays and microscopy and the presence of flagella proteins was determined by SDS-PAGE once isolated from 24 h overnight liquid cultures grown at 22°C as previously described [20], [79].

### SDS-PAGE and protein sequencing

Proteins present in 10 ml of cell-free supernatant taken from *Y. pseudotuberculosis* QS and motility mutants grown to the same OD_600_ (overnight in YLB at 22°C, 28°C and 37°C) were concentrated by trichloroacetic acid precipitation, subjected to SDS-PAGE and the relevant bands excised. After in-gel tryptic digestion, the resulting peptides were identified by matrix-assisted laser desorption ionization-time of flight (MALDI-TOF)-MS sequencing as previously described [20].

## Supporting Information

Figure S1 Orthogonal images of Figure 1A showing the *Y. pseudotuberculosis* YpIII biofilm depth in cross section through the x and Y planes (A). Examples of severity level 1 and 2 biofilms on the surface of *C. elegans* are shown in (B and C).
(0.54 MB TIF)

Figure S2 Protein profiles of supernatants taken from *Y. pseudotuberculosis* YpIII and the QS mutants grown at 37°C. Four up-regulated proteins were identified as YopM/H, LcrV, YopN and FliC.
(0.15 MB TIF)

Table S1 Strains used in this study.
(0.08 MB DOC)

Table S2 Plasmids used in this study.
(0.11 MB DOC)

Table S3 Primers used in this study.
(0.04 MB DOC)

## References

[1] Navarro L, Alto NM, Dixon JE (2005). Functions of the Yersinia effector proteins in inhibiting host immune responses.. Curr Opin Microbiol.

[2] Cornelis GR (2002). Yersinia type III secretion: send in the effectors.. J Cell Biol.

[3] Galan JE, Wolf-Watz H (2006). Protein delivery into eukaryotic cells by type III secretion machines.. Nature.

[4] Ramamurthi KS, Schneewind O (2002). Type III protein secretion in Yersinia species.. Annu Rev Cell Dev Biol.

[5] Darby C, Hsu JW, Ghori N, Falkow S (2002). *Caenorhabditis elegans* - Plague bacteria biofilm blocks food intake.. Nature.

[6] Joshua GWP, Karlyshev AV, Smith MP, Isherwood KE, Titball RW (2003). A Caenorhabditis elegans model of Yersinia infection: biofilm formation on a biotic surface.. Microbiology-Sgm.

[7] Hinnebusch BJ, Perry RD, Schwan TG (1996). Role of the *Yersinia pestis* hemin storage (*hms*) locus in the transmission of plague by fleas.. Science.

[8] Stoodley P, Sauer K, Davies DG, Costerton JW (2002). Biofilms as complex differentiated communities.. Annu Rev Microbiol.

[9] Jarrett CO, Deak E, Isherwood KE, Oyston PC, Fischer ER (2004). Transmission of *Yersinia pestis* from an infectious biofilm in the flea vector.. J Infect Dis.

[10] Platt HM, Lorenzen S (1994). Forward in Phylogenetic systematics of free-living nematodes..

[11] Matz C, Kjelleberg S (2005). Off the hook - how bacteria survive protozoan grazing.. Trends Microbiol.

[12] Tan L, Darby C (2004). A movable surface: Formation of Yersinia sp biofilms on motile *Caenorhabditis elegans*.. J Bacteriol.

[13] Salmond GPC, Bycroft BW, Stewart GSAB, Williams P (1995). The bacterial enigma-cracking the code of cell-cell communication.. Mol Microbiol.

[14] Williams P, Cámara M, Hardman A, Swift S, Milton D (2000). Quorum sensing and the population-dependent control of virulence.. Philos T Roy Soc B.

[15] Swift S, Downie JA, Whitehead NA, Barnard AML, Salmond GPC, Williams P (2001). Quorum sensing as a population-density-dependent determinant of bacterial physiology.. Adv Microb Phys.

[16] Cámara M, Williams P, Hardman A (2002). Controlling infection by tuning in and turning down the volume of bacterial small-talk.. Lancet Infect Dis.

[17] Williams P, Winzer K, Chan WC, Cámara M (2007). Look who's talking: communication and quorum sensing in the bacterial world.. Philos T Roy Soc B.

[18] Atkinson S, Throup JP, Stewart GSAB, Williams P (1999). A hierarchical quorum sensing system in *Yersinia pseudotuberculosis* is involved in the regulation of motility and clumping.. Mol Microbiol.

[19] Atkinson S, Chang CY, Patrick HL, Buckley CMF, Wang Y (2008). Functional interplay between the *Yersinia pseudotuberculosis* YpsRI and YtbRI quorum sensing systems modulates swimming motility by controlling expression of *flhDC* and *fliA*.. Mol Microbiol.

[20] Atkinson S, Chang CY, Sockett RE, Cámara M, Williams P (2006). Quorum sensing in *Yersinia enterocolitica* controls swimming and swarming motility.. J Bacteriol.

[21] Kirwan JP, Gould TA, Schweizer HP, Bearden SW, Murphy RC (2006). Quorum-sensing signal synthesis by the *Yersinia pestis* acyl homoserine lactone synthase YspI.. J Bacteriol.

[22] Swift S, Isherwood KE, Atkinson S, Oyston P, Stewart GSAB, England R, Hobbs G, Bainton NJ, Roberts DM (1999). Quorum sensing in *Aeromonas* and *Yersinia*.. Microbial Signalling and Communication.

[23] Isherwood EK (2001). Quorum sensing in *Yersinia pestis*.. PhD thesis.

[24] Young GM, Carniel E, Hinnebusch BJ (2004). Flagella:Organelles for motility and protein secretion.. Yersinia molecular and cellular biology.

[25] Charlton TS, de Nys R, Netting A, Kumar N, Hentzer M (2000). A novel and sensitive method for the quantification of *N*-3-oxoacyl homoserine lactones using gas chromatography-mass spectrometry: application to a model bacterial biofilm.. Environ Microbiol.

[26] Lynch MJ, Swift S, Kirke DF, Keevil CW, Dodd CER (2002). The regulation of biofilm development by quorum sensing in *Aeromonas hydrophila*.. Environ Microbiol.

[27] Atkinson S, Cámara M, Williams P, Kjellberg S, Givskov M (2007). *N*-Acylhomoserine lactones, quorum sensing and biofilm development in Gram-negative bacteria.. The biofilm mode of life. Mechanisms and adaptations.

[28] Bjarnsholt T, Jensen PO, Burmolle M, Hentzer M, Haagensen JAJ (2005). *Pseudomonas aeruginosa* tolerance to tobramycin, hydrogen peroxide and polymorphonuclear leukocytes is quorum-sensing dependent.. Microbiology-Sgm.

[29] Jensen PO, Bjarnsholt T, Phipps R, Rasmussen TB, Calum H (2007). Rapid necrotic killing of polymorphonuclear leukocytes is caused by quorum sensing-controlled production of rhamnolipid by *Pseudomonas aeruginosa*.. Microbiology-Sgm.

[30] Allesen-Holm M, Barken KB, Yang L, Klausen M, Webb JS (2006). A characterization of DNA release in *Pseudomonas aeruginosa* cultures and biofilms.. Mol Microbiol.

[31] McClean KH, Winson MK, Fish L, Taylor A, Chhabra SR (1997). Quorum sensing and *Chromobacterium violaceum*: exploitation of violacein production and inhibition for the detection of *N*-acylhomoserine lactones.. Microbiology-Uk.

[32] Andersen JB, Heydorn A, Hentzer M, Eberl L, Geisenberger O (2001). *gfp*-based *N*-acyl homoserine-lactone sensor systems for detection of bacterial communication.. Appl Environ Microbiol.

[33] Roche DM, Byers JT, Smith DS, Glansdorp FG, Spring DR (2004). Communications blackout? Do *N*-acylhomoserine lactone-degrading enzymes have any role in quorum sensing?. Microbiology-Sgm.

[34] Young GM, Schmiel DH, Miller VL (1999). A new pathway for the secretion of virulence factors by bacteria: The flagellar export apparatus functions as a protein-secretion system.. Proc Natl Acad Sci U S A.

[35] Saijo-Hamano Y, Imada K, Minamino T, Kihara M, Shimada M (2010). Structure of the cytoplasmic domain of FlhA and implication for flagellar type III protein export.. Mol Microbiol.

[36] Silva-Herzog E, Ferracci F, Jackson MW, Joseph SS, Plano GV (2008). Membrane localization and topology of the *Yersinia pestis* YscJ lipoprotein.. Microbiology-Sgm.

[37] Marenne MN, Mota LJ, Cornelis GR, Carniel E, Hinnebusch BJ (2004). The pYV plasmid and the Ysc-Yop Type III secretion system.. Yersinia molecular and cellular biology.

[38] O'Toole G, Kaplan HB, Kolter R (2000). Biofilm formation as microbial development.. Annu Rev Microbiol.

[39] Kjelleberg S, Molin S (2002). Is there a role for quorum sensing signals in bacterial biofilms?. Curr Opin Microbiol.

[40] Sauer K, Camper AK, Ehrlich GD, Costerton JW, Davies DG (2002). *Pseudomonas aeruginosa* displays multiple phenotypes during development as a biofilm.. J Bacteriol.

[41] Klausen M, Aes-Jorgensen A, Molin S, Tolker-Nielsen T (2003). Involvement of bacterial migration in the development of complex multicellular structures in *Pseudomonas aeruginosa* biofilms.. Mol Microbiol.

[42] Webb JS, Thompson LS, James S, Charlton T, Tolker-Nielsen T (2003). Cell death in *Pseudomonas aeruginosa* biofilm development.. J Bacteriol.

[43] Tan L, Darby C (2006). *Yersinia pestis* YrbH is a multifunctional protein required for both 3-deoxy-D-manno-oct-2-ulosonic acid biosynthesis and biofilm formation.. Mol Microbiol.

[44] Sizemore RK, Caldwell JJ, Kendrick AS (1990). Alternate Gram staining technique using a fluorescent lectin.. Appl Environ Microbiol.

[45] Bobrov AG, Kirillina O, Forman S, Mack D, Perry RD (2008). Insights into *Yersinia pestis* biofilm development: topology and co-interaction of Hms inner membrane proteins involved in exopolysaccharide production.. Environmental Microbiology.

[46] Drace K, Darby C (2008). The *hmsHFRS* operon of *Xenorhabdus nematophila* is required for biofilm attachment to *Caenorhabditis elegans*.. Appl Environ Microbiol.

[47] Sun YC, Hinnebusch BJ, Darby C (2008). Experimental evidence for negative selection in the evolution of a *Yersinia pestis* pseudogene.. Proc Natl Acad Sci U S A.

[48] Sun YC, Koumoutsi A, Darby C (2009). The response regulator PhoP negatively regulates *Yersinia pseudotuberculosis* and *Yersinia pestis* biofilms.. FEMS Microbiol Lett.

[49] Kirillina O, Fetherston JD, Bobrov AG, Abney J, Perry RD (2004). HmsP, a putative phosphodiesterase, and HmsT, a putative diguanylate cyclase, control Hms-dependent biofilm formation in *Yersinia pestis*.. Mol Microbiol.

[50] Simm R, Fetherston JD, Kader A, Romling U, Perry RD (2005). Phenotypic convergence mediated by GGDEF-domain-containing proteins.. J Bacteriol.

[51] Bobrov AG, Kirillina O, Perry RD (2007). Regulation of biofilm formation in *Yersinia pestis*.. Adv Exp Med Biol.

[52] Jacobi CA, Bach A, Eberl L, Steidle A, Heesemann J (2003). Detection of *N*-(3-oxohexanoyl)-L-homoserine lactone in mice infected with *Yersinia enterocolitica* serotype O8.. Infect Immun.

[53] Pratt LA, Kolter R (1998). Genetic analysis of *Escherichia coli* biofilm formation: roles of flagella, motility, chemotaxis and type I pili.. Mol Microbiol.

[54] O'Toole GA, Kolter R (1998). Flagellar and twitching motility are necessary for *Pseudomonas aeruginosa* biofilm development.. Mol Microbiol.

[55] Hossain MM, Tsuyumu S (2006). Flagella-mediated motility is required for biofilm formation by *Erwinia carotovora* subsp. *carotovora*.. J Gen Plant Pathol.

[56] Kim TJ, Young BM, Young GM (2008). Effect of flagellar mutations on *Yersinia enterocolitica* biofilm formation.. Appl Environ Microbiol.

[57] Gomez-Gomez L, Boller T (2002). Flagellin perception: a paradigm for innate immunity.. Trends in Plant Sci.

[58] Bleves S, Marenne MN, Detry G, Cornelis GR (2002). Up-regulation of the *Yersinia enterocolitica* yop regulon by deletion of the flagellum master operon *flhDC*.. J Bacteriol.

[59] T, Wattiau P, Brasseur R, Ruysschaert JM, Cornelis G (1990). Secretion of Yop Proteins by Yersiniae.. Infect Immun.

[60] Brubaker RR, Surgalla MJ (1964). Effect of Ca^2+^ and Mg^2+^ on lysis growth and production of virulence antigens.. J Infect Dis.

[61] Bolin I, Wolf-Watz H (1984). Molecular cloning of the temperature inducible outer membrane protein-1 of *Yersinia pseudotuberculosis*.. Infect Immun.

[62] Bolin I, Portnoy DA, Watz HW (1985). Expression of the Temperature inducible outer membrane proteins of Yersiniae.. Infect Immun.

[63] Forsberg A, Viitanen AM, Skurnik M, Wolf-Watz H (1991). The surface located YopN protein is involved in calcium signal transduction in *Yersinia pseudotuberculosis*.. Mol Microbiol.

[64] Drace K, McLaughlin S, Darby C (2009). *Caenorhabditis elegans* BAH-1 is a DUF23 protein expressed in seam cells and required for microbial biofilm binding to the cuticle.. Plos One.

[65] Darby C, Chakraborti A, Politz SM, Daniels CC, Tan L (2007). *Caenorhabditis elegans* mutants resistant to attachment of Yersinia biofilms.. Genetics.

[66] Qazi SNA, Rees CED, Mellits KH, Hill PJ (2001). Development of *gfp* vectors for expression in *Listeria monocytogenes* and other low G+C Gram-positive bacteria.. Microb Ecol.

[67] Lennox ES (1955). Transduction of linked genetic characters of the host by bacteriophage P1.. Virology.

[68] Yates EA, Philipp B, Buckley C, Atkinson S, Chhabra SR (2002). *N*-acylhomoserine lactones undergo lactonolysis in a pH-, temperature-, and acyl chain length-dependent manner during growth of *Yersinia pseudotuberculosis* and *Pseudomonas aeruginosa*.. Infect Immun.

[69] Riley G, Toma S (1989). Detection of pathogenic *Yersinia enterocolitica* by using congo red-magnesium oxalate agar medium.. J Clin Microbiol.

[70] Lewis JA, Fleming TJ (1995). *Caenorhabditis elegans*: Modern biological analysis of an organism..

[71] Tarr SAJ (1972). The assesment of disease incidence and crop loss..

[72] Vilain S, Pretorius JM, Theron J, Brozel VS (2009). DNA as an adhesin: *Bacillus cereus* requires extracellular DNA to form biofilms.. Appl Environ Microbiol.

[73] Bartolome B, Jubete Y, Martinez E, Delacruz F (1991). Construction and properties of a family of pACYC184-derived cloning vectors compatible with pBR322 and its derivatives.. Gene.

[74] Brenner S (1974). Genetics of *Caenorhabditis elegans*.. Genetics.

[75] Derbise A, Lesic B, Dacheux D, Ghigo JM, Carniel E (2003). A rapid and simple method for inactivating chromosomal genes in Yersinia.. FEMS Immunol Med Microbiol.

[76] Chain PSG, Carniel E, Larimer FW, Lamerdin J, Stoutland PO (2004). Insights into the evolution of *Yersinia pestis* through whole genome comparison with *Yersinia pseudotuberculosis*.. Proc Natl Acad Sci U S A.

[77] Yanischperron C, Vieira J, Messing J (1985). Improved M13 phage cloning vectors and host strains - nucleotide-sequences of the M13, Mp18 and pUC19 vectors.. Gene.

[78] Stewart GSAB, Lubinskymink S, Jackson CG, Cassel A, Kuhn J (1986). pHG165-A pBR322 Copy Number Derivative of pUC8 for Cloning and Expression.. Plasmid.

[79] Sockett RE, Williams P, Salmond G, Ketley JM (1998). Characterising Flagella and Motile Behaviour.. Methods in microbiology: methods for studying pathogenic bacteria.

