# Supplementary figures and images for: Biofilm Development on Caenorhabditis elegans by Yersinia Is Facilitated by Quorum Sensing-Dependent Repression of Type III Secretion
Source: PLoS Pathog. 2011 Jan 6;7(1):e1001250. doi: 10.1371/journal.ppat.1001250 (PMC3017118; doi:10.1371/journal.ppat.1001250)

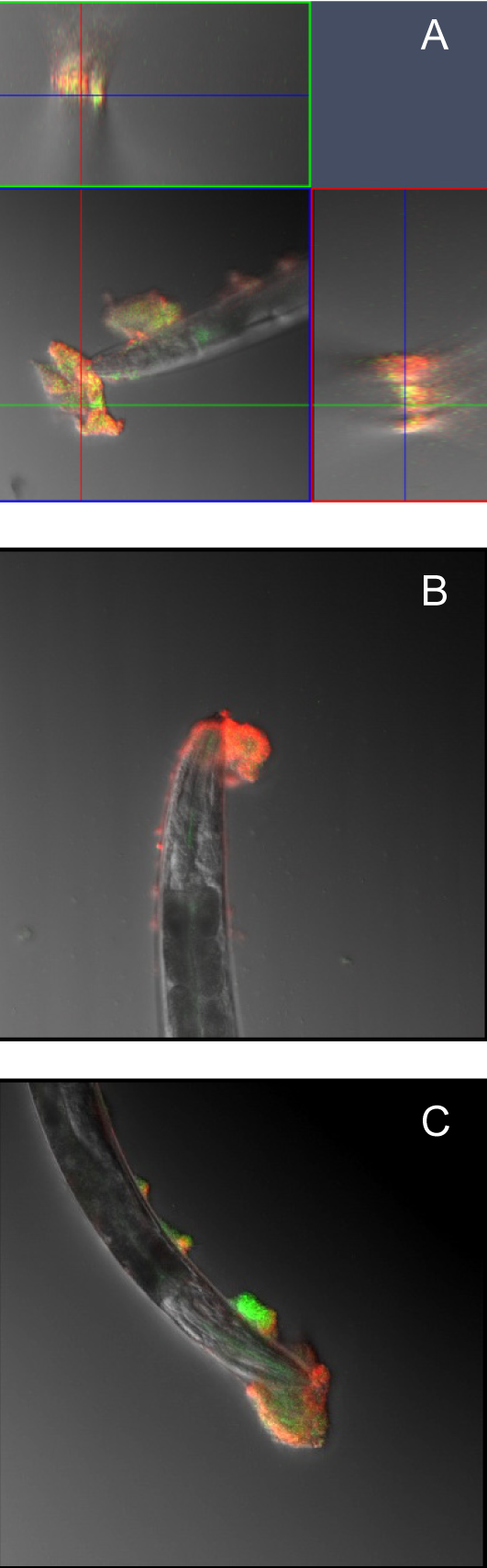

Supplement: Figure S1 — Orthogonal images of Figure 1A showing the Y. pseudotuberculosis YpIII biofilm depth in cross section through the x and Y planes (A). Examples of severity level 1 and 2 biofilms on the surface of C. elegans are shown in (B and C). (0.54 MB TIF) [file ppat.1001250.s001.tif]

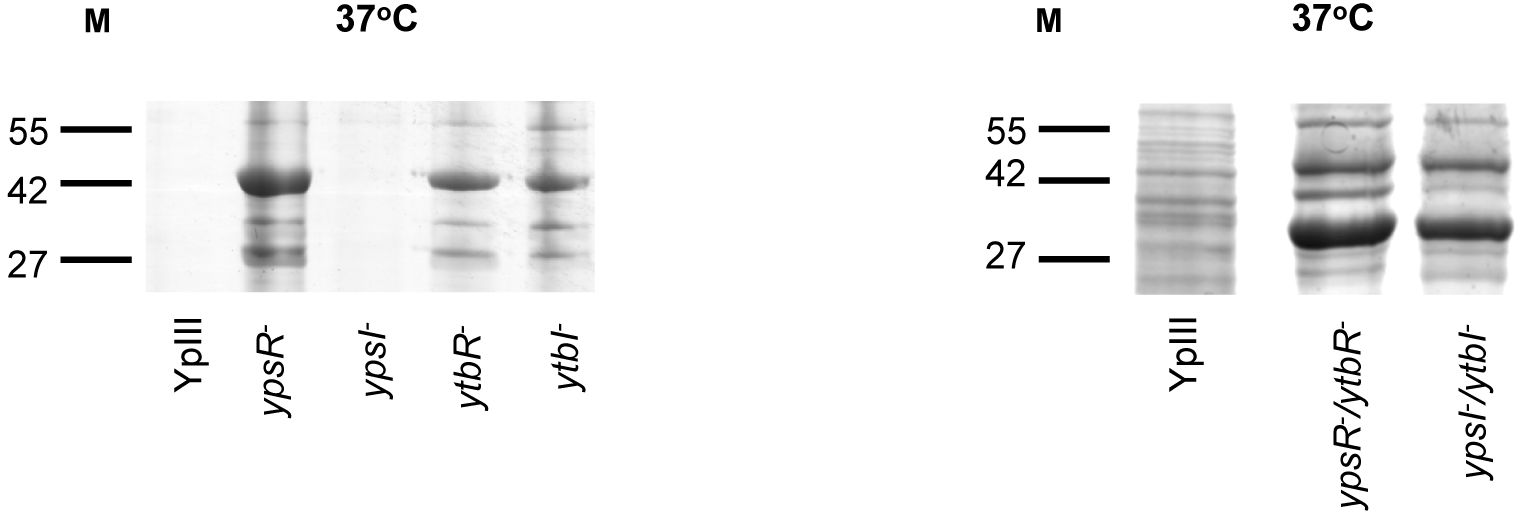

Supplement: Figure S2 — Protein profiles of supernatants taken from Y. pseudotuberculosis YpIII and the QS mutants grown at 37°C. Four up-regulated proteins were identified as YopM/H, LcrV, YopN and FliC. (0.15 MB TIF) [file ppat.1001250.s002.tif]
