# Supplementary material for: Biofilm Development on Caenorhabditis elegans by Yersinia Is Facilitated by Quorum Sensing-Dependent Repression of Type III Secretion
Source: PLoS Pathog. 2011 Jan 6;7(1):e1001250. doi: 10.1371/journal.ppat.1001250 (PMC3017118; doi:10.1371/journal.ppat.1001250)
Supplement: Table S1 — Strains used in this study. (0.08 MB DOC) [file ppat.1001250.s003.doc]

| **Strain** | **Description** | **Reference/**  **Source** |
| --- | --- | --- |
| ***Escherichia coli*** | | |
| OP50 | Uracil auxotroph which serves as a nutrient source for *C. elegans* and as a control for uninfected worms. Tc^R^. | [1] |
| DH5-α | *E. coli* K-12 cloning strain. [F φ80d*lacZ*ΔM15Δ (*lacZYA-argF*) U169 *recAI endAI hsdR17* (r_K_- m_K_^+^) *supE44 thiI gyrI relAI*]. | [2] |
| S-17 λ*pir* | λ *-pir* lysogen of S17-1 [*thi pro hsdR*– *hsd*M+ *recA* RP4 2-Tc::Mu-Km::Tn*7*]. Permissive host capable if transferring suicide plasmids requiring the Pir protein by conjugation to recipient cells. Sm^R^. | [3] |
| ***Chromobacterium***  ***Violaceum*** | | |
| CV026 | White *cviI* mutant generated from ATCC 31532 which produces the purple pigment violacein in the presence of AHLs. | [4] |
| ***Yersinia***  ***Pseudotuberculosis*** | | |
| YpIII pIB1 | Parent strain of YpIII harbouring the virulence plasmid pYV. Serotype O:3. Nal^R^. | [5] |
| YpIII *flhDC* | YpIII containing a mutation in the motility master regulator *flhDC*. Tc^R^. | [6] |
| YpIII *ypsI* | YpIII *ypsI* AHL synthase mutant. Km^R^. | [7] |
| YpIII *ypsR* | YpIII *ypsR* AHL response regulator mutant. Km^R^. | [7] |
| YpIII *ytbI* | YpIII *ytbI* AHL synthase mutant. Cm^R^. | [6] |
| YpIII *ytbR* | YpIII *ytbR* AHL response regulator mutant. Cm^R^. | [6] |
| YpIII *ypsI/ytbI* | YpIII *ypsI/ytbI* AHL synthase double mutant. Cm^R^ Km^R^. | [6] |
| YpIII *ypsR/ytbR* | YpIII *ypsR/ytbR* AHL response regulator double mutant. Cm^R^ Km^R^. | [6] |
| YpIII *ypsI/ytbI*  pSA291 | YpIII *ypsI/ytbI* AHL synthase double mutant harbouring *ypsI* and *ytbI* on the complementation vector pSA291. Cm^R^ Km^R^ Ap^R^. | [6] |
| YpIII *flhDC* pSA220 | YpIII *flhDC* mutant harbouring *flhDC* on the complement plasmid pSA220. Tc^R^ Ap^R^. | [6] |
| YpIII *ypsI/ytbI* pSB2020 | YpIII *ypsI/ytbI* AHL synthase double mutant constitutively expressing *gfp* from pSB2020. Cm^R^ Km^R^ Ap^R^. | This study |
| YpIII *flhDC* pYV^-^ | YpIII *flhDC* mutant cured of the pYV virulence plasmid. Tc^R^. | This study |
| YpIII *fliA* | YpIII containing a mutation in the flagella sigma factor, *fliA*. Km^R^. | This study |
| YpIII *flhA* | YpIII containing a mutation in the flagella structural gene, *flhA*. Km^R^ | This study |
| YpIII *fliC* | YpIII containing a mutation in the flagella filament gene, *fliC*. Km^R^ | This study |
|  |  |  |
| YpIII *ypsI/ytbI*  pJBA89 | YpIII *ypsI/ytbI* AHL synthase double mutant harbouring *gfp3* expressed in an AHL dependent manner on pJBA89. Cm^R^ Km^R^ Ap^R^. | This study |
| YpIII *ypsI/ytbI* pYV^-^ | YpIII *ypsI/ytbI* AHL synthase double mutant cured of the pYV virulence plasmid. Cm^R^ Km^R^. | This study |
|  |  |  |
| YpIII *ypsI/ytbI/yscJ* | Deletion of the *yscJ* type three secretion system inner ring component in the *ypsI/ytbI* double AHL synthase mutant background. Cm^R^ Km^R^ Tc^R^. | This study |
| YpIII *ypsI/ytbI/yscJ* pSB2020 | YpIII *ypsI/ytbI/yscJ* triple mutant constitutively expressing *gfp* from pSB2020 Cm^R^ Km^R^ Ap^R^ | This study |
| YpIII *ypsI/ytbI/yscJ* pHG::*yscJ* | YpIII *ypsI/ytbI/yscJ* triple mutant harbouring *yscJ* on pHG::*yscJ* for complementation. Cm^R^ Km^R^ Ap^R^. | This study |
| ***Caenorhabditis***  ***elegans*** | | |
| N2 Bristol | Wild-type *C. elegans*. Obtained from the The Caenorhabditis Genetics Centre, University of Minnesota. | [1] |

Reference List

1. Lewis, J. A. and Fleming, J. T. (1995) *Caenorhabditis elegans*: Modern Biological Analysis of an Organism. New York: Academic Press. 39 p.

2. Hanahan D (1983) Studies on transformation of *Escherichia coli* with plasmids. J Mol Biol 166: 557-580.

3. Simon R, Priefer U, Puhler A (1983) A broad host range mobilization system for invivo genetic engineering transposon mutagenesis in gram negative bacteria. Biotechnology 1: 784-791.

4. McClean KH, Winson MK, Fish L, Taylor A, Chhabra SR, Camara M, *et al.* (1997) Quorum sensing and *Chromobacterium violaceum*: exploitation of violacein production and inhibition for the detection of *N*- acylhomoserine lactones. Microbiology-Uk 143: 3703-3711.

5. Rosqvist R, Skurnik M, Wolfwatz H (1988) Increased virulence of *Yersinia pseudotuberculosis* by two independent mutations. Nature 334: 522-525.

6. Atkinson S, Chang CY, Patrick HL, Buckley CMF, Wang Y, *et al.* (2008) Functional interplay between the *Yersinia pseudotuberculosis* YpsRI and YtbRI quorum sensing systems modulates swimming motility by controlling expression of *flhDC* and *fliA*. Mol Microbiol 69: 137-151.

7. Atkinson S, Throup JP, Stewart GSAB, Williams P (1999) A hierarchical quorum-sensing system in *Yersinia pseudotuberculosis* is involved in the regulation of motility and clumping. Mol Microbiol 33: 1267-1277.
