# Supplementary material for: Biofilm Development on Caenorhabditis elegans by Yersinia Is Facilitated by Quorum Sensing-Dependent Repression of Type III Secretion
Source: PLoS Pathog. 2011 Jan 6;7(1):e1001250. doi: 10.1371/journal.ppat.1001250 (PMC3017118; doi:10.1371/journal.ppat.1001250)
Supplement: Table S2 — Plasmids used in this study. (0.11 MB DOC) [file ppat.1001250.s004.doc]

| **Plasmid** | **Description** | **Reference/**  **Source** |
| --- | --- | --- |
|  |  |  |
| pGEMT/easy | PCR product cloning vector. Ap^R^. | Promega |
| pBluescript SKII+ | Cloning vector, ColE1 replicon. Ap^R^. | Stratagene |
| pDM4 | Cm^R^ suicide vector for chromosomal insertion, mobRK2, oriR6K (*pir* requiring). *sacBR* of *Bacillus subtilis*. | [1] |
| pHG327 | Low-copy number complementation vector Ap^R^. | [2] |
| pSB2020 | constitutively expresses *gfp3*. Ap^R^. | [3] |
| pJBA89 | AHL biosensor which reports as Gfp the presence of AHLs. | [4] |
| pSU18 | Cloning vector | [5] |
| pUC4K | Source of kanamycin resistance cassette | Pharmacia |
| pBR322 | Tetracycline cassette origin | [6] |
| pSA236 | pSU18 containing *aiiA*, capable of degrading AHLs. Cm^R^. | [7] |
| pBlue-tet | Source of a tetracycline cassette amplified from pBR322 and cloned into pBluescript II KS+. Tet^R^ Ap^R^. | This study |
| pfliC | *fliC* PCR product cloned into pGEMT/Easy Ap^R^ | This study |
| pDM *fliC*-Km | pDM4 containing *fliC* into which has been inserted the Km^R^ gene to replace the central *fliC* sequence. Cm^R^ Km^R^. | This study |
| pBlue::*yscJ* | *yscJ* PCR product cloned into pBluescript SKII+ Ap^R^ | This study |
| pHG::*yscJ* | *yscJ* cloned into pHG327 as a *kpn*I *pst*I fragment Ap^R^ | This study |
| pSA220 | *flhDC* PCR product cloned into pGEMT/Easy Ap^R^ | This study |

Reference List

1. OToole R, Milton DL, Wolfwatz H (1996) Chemotactic motility is required for invasion of the host by the fish pathogen *Vibrio anguillarum*. Mol Microbiol 19: 625-637.

2. Stewart GSAB, Lubinskymink S, Jackson CG, Cassel A, Kuhn J (1986) pHG165 - A pBR322 copy number derivative of pUC8 for cloning and expression. Plasmid 15: 172-181.

3. Qazi SNA, Rees CED, Mellits KH, Hill PJ (2001) Development of gfp vectors for expression in *Listeria monocytogenes* and other low G+C gram positive bacteria. Microb Ecol 41: 301-309.

4. Andersen JB, Heydorn A, Hentzer M, Eberl L, Geisenberger O, *et al.* (2001) gfp-based *N*-acyl homoserine-lactone sensor systems for detection of bacterial communication. Appl Environ Microbiol 67: 575-585.

5. Bartolome B, Jubete Y, Martinez E, Delacruz F (1991) Construction and properties of a family of pACYC184-derived cloning vectors compatible with pBR322 and its derivatives. Gene 102: 75-78.

6. Bolivar F, Rodriguez RL, Greene PJ, Betlach MC, Heyneker HL, *et al.* (1977) Construction and characterization of new cloning vehicles II. A multipurpose cloning system. Gene 2: 95-113.

7. Atkinson S, Chang CY, Patrick HL, Buckley CMF, Wang Y, *et al*. (2008) Functional interplay between the *Yersinia pseudotuberculosis* YpsRI and YtbRI quorum sensing systems modulates swimming motility by controlling expression of flhDC and fliA. Mol Microbiol 69: 137-151.
