# Supplementary material for: Biofilm Development on Caenorhabditis elegans by Yersinia Is Facilitated by Quorum Sensing-Dependent Repression of Type III Secretion
Source: PLoS Pathog. 2011 Jan 6;7(1):e1001250. doi: 10.1371/journal.ppat.1001250 (PMC3017118; doi:10.1371/journal.ppat.1001250)
Supplement: Table S3 — Primers used in this study. (0.04 MB DOC) [file ppat.1001250.s005.doc]

| Primer Name | Sequence |
| --- | --- |
| fliA1up-F | gatgctgattacgcgactg |
| fliA1up-R | gattttgagacacaacgtggctttcattcttatccatcacgc |
| fliA1down-F | gatgctcgatgagtttttctaacgcgtcagtcaacttcatag |
| fliA1down-R | cagcgccgtcagttcttcc |
| km-F | gaaagccacgttgtgtctcaa |
| km-R | ttagaaaaactcatcgagcat |
| flhA1up-F | gaaggcgacccacatgtg |
| flhA1up-R | gagacacaacgtggctttcgattggcccagcgagaatttgcc |
| flhA1down-F | catggtgctgggtatccg |
| flhA1down-R | gctcgatgagtttttctaagcattattggcccgcttcttgcgc |
| DC1 | gatggctggaatagcagg |
| DC2 | cttgaagtgcattctcagc |
| FlhDF | cacactagtgtgctaacg |
| FlhCR | ccttctagaacgagataac |
| aiiA-F | atgacagtaaagaagctt |
| aiiA-R | aagggacttatatatatc |
| YscJaFor | ctgaattgcgtagtgtattgcagcag |
| YscJupR-Tet | gagcgcattgttagatttcattagttttcacccccccttcga |
| YscJdownF-tet | gagccgggccacctcgacctgacgtaacacgagcatactgtc |
| YscJbRev | ccgcaagcgagcgagagattac |
| TetFor | atgaaatctaacaatgcgctc |
| TetRev | tcaggtcgaggtggcccggctc |
